# Markers of adipose tissue hypoxia are elevated in subcutaneous adipose tissue of severely obese patients with obesity hypoventilation syndrome but not in the moderately obese

**DOI:** 10.1038/s41366-021-00793-7

**Published:** 2021-03-23

**Authors:** Marijana Todorčević, Ari R. Manuel, Luke Austen, Zoi Michailidou, Jonathan M. Hazlehurst, Matt Neville, John R. Stradling, Fredrik Karpe

**Affiliations:** 1 Oxford Centre for Diabetes, Endocrinology and Metabolism, Radcliffe Department of Medicine, University of Oxford, Oxford, UK; 2 Oxford Respiratory Trials Unit, Churchill Hospital, University of Oxford, Oxford, UK; 3 Liverpool Centre for Respiratory Science, University of Liverpool, Liverpool, UK; 4 Queen’s Medical Research, Institute Centre for Cardiovascular Research, University of Edinburgh, Edinburgh, UK; 5 Institute of Metabolism and Systems Research, University of Birmingham, Birmingham, UK; 6 NIHR Oxford Biomedical Research Centre, OUH Trust, Churchill Hospital, Oxford, UK

**Keywords:** Fatty acids, Obesity

## Abstract

It has been suggested that metabolic dysfunction in obesity is at least in part driven by adipose tissue (AT) hypoxia. However, studies on AT hypoxia in humans have shown conflicting data. Therefore we aimed to investigate if markers of AT hypoxia were present in the subcutaneous AT of severly obese individuals (class III obesity) with and without hypoventilation syndrome (OHS) in comparison to moderately obese (class I obesity) and lean controls. To provide a proof-of-concept study, we quantified AT hypoxia by hypoxia inducible factor 1 A (HIF1A) protein abundance in human participants ranging from lean to severly obese (class III obesity). On top of that nightly arterial O_2_ saturation in individuals with obesity OHS was assessed. Subjects with class III obesity (BMI > 40 kg/m^2^) and OHS exhibited significantly higher adipose HIF1A protein levels versus those with class I obesity (BMI 30–34.9 kg/m^2^) and lean controls whereas those with class III obesity without OHS showed an intermediate response. *HIF1A* gene expression was not well correlated with protein abundance. Although these data demonstrate genuine AT hypoxia in the expected pathophysiological context of OHS, we did not observe a hypoxia signal in lesser degrees of obesity suggesting that adipose dysfunction may not be driven by hypoxia in moderate obesity.

## Introduction

Although tissue hypoxia can be caused by low oxygen delivery, it can be augmented by high oxygen consumption. A general feature of human white AT is its low oxygen consumption, a high degree of glycolysis and a respiratory quotient close to 1.0, indicating a near absence of O_2_-consuming fatty acid oxidation [1]. However, there may well be species differences as artificially reducing the tissue oxygen consumption in mice, through adipose-specific ablation of adenine nucleotide translocase 2 (ablates mitochondrial fatty acid oxidation) preserves higher oxygen tension in AT with normalised whole body metabolic function [2].

Methods to verify hypoxia in AT is in vivo include staining with pimonidazole, detection of HIF1A protein induction or direct monitoring oxygen tension in situ. However, these approaches have given rise to conflicting data with signs of AT hypoxia [3] or increased [4] O_2_ tension. Therefore, in order to further evaluate the presence of hypoxia in human AT, we assembled participants with a wide range of body fatness, also including a subgroup with obesity hypoventilation syndrome (OHS). The presence of HIF1A protein in AT biopsies together with downstream transcriptional targets of HIF1A were taken as indicators of tissue hypoxia.

## Methods

### Subcutaneous abdominal adipose biopsies from patients of different levels of adiposity with and without OHS

Eight lean (mean BMI 24.2 kg/m^2^, six men) and 8 class I obese (mean BMI 32.5 kg/m^2^, six men) healthy participants were recruited from the Oxford Biobank [5]. Subcutaneous AT biopsies were taken under local anaesthesia using a liposuction method first thing in the morning and immediately snap frozen in liquid N_2_. Two further groups (*n* = 9, six men in each group) of class III obese patients with or without OHS (both groups with mean BMI of 45.3 kg/m^2^) were identified from a previous study [6] but from the same geographic and demographic region as the Oxford Biobank, and provided a biopsy using the same technique. None of the participants had a formal diagnosis of type 2 diabetes. The lean and class I obese groups were not monitored overnight with pulse oximetry (SpO_2_) but were questioned of symptoms related to OHS, which were negated. The class III obese groups had pulse oximetry (SpO_2_) during the night preceding the biopsy. The oxygen desaturation index, which is the number of oxygen desaturations (by ≥4% from the average saturations for the preceding 120 s that last >10 s) per h of study and second the proportion of the sleeping time spent below SpO_2_ of 90% (Table 1) were measured. The latter index was used to dichotomise the class III obese OHS vs non-OHS group (Table 1).

**Table 1:** General characteristics of the participants in the groups.

Group	*N*	Sex (m/f)	BMI (kg/m^2^)	WHR	Proportion of sleeping time spent <90% SpO_2_	HbA1c (%)	Fasting glucose (mmol/l)	Fasting insulin (pmol/l)	TAG (mmol/l)	HDL (mmol/l)
Lean	8	6/2	24.2 ± 0.3	0.87 ± 0.03	–	–	5.2 ± 0.1	64 ± 12	0.96 ± 0.11	1.54 ± 0.08
Class I Obesity	8	6/2	32.5 ± 0.3*	0.96 ± 0.02*	–	–	5.2 ± 0.2	92 ± 12*	1.16 ± 0.15	1.15 ± 0.11*
Class III Obesity	9	6/3	45.3 ± 2.9*	1.02 ± 0.04*	3.2 ± 0.5	5.9 ± 0.2	5.3 ± 0.2	203 ± 17*	1.35 ± 0.19*	–
Class III Obesity with OHS	9	6/3	45.3 ± 3.3*	0.96 ± 0.08*	58.7 ± 7.5	6.6 ± 0.3	5.5 ± 0.3	228 ± 43*	1.97 ± 0.39*	–

Averaged data are mean ± SEM. (*<0.05 vs lean control).

### Isolation of protein from human AT and WB for HIF1A

The frozen AT specimens were homogenised in a urea/SDS buffer supplemented with Complete Protease Inhibitor (Roche Applied Science). Samples (80 μg total protein) were run on a 7% acrylamide gel and western blotting (WB) was conducted as previously described [7]. Ten samples were run per gel with groups mixed. A rabbit anti-mouse HIF-1α Primary antibody (10006421 Cayman) and HRP-conjugated anti-β-actin (Abcam) was used for detection. Densitometry was performed using ImageJ, and the sample HIF1A abundance was corrected for actin. Due to technical difficulties of achieving high quality and quantifiable western blot images, samples had to be re-run on several occasion which led to sample depletion. For this reason the final number of successful quantifications was less than total participants. Gene expression in adipose tissue samples was determined as described [8].

### HIF1A in human primary adipocytes

As a proof-of-concept study to observe the upregulation of HIF1A in response to hypoxia in adipocytes, primary human preadipocytes were derived from subcutaneous abdominal AT and differentiated as described [9]. Fully differentiated adipocytes were exposed to normoxic or hypoxic conditions for 24 h in three separate hypoxia chambers (InVivO_2_ 400, Baker Ruskinn, UK) containing 0.1%, 1% or 5% O_2_, all with 5% CO_2,_ while the control cells were cultured in a standard incubators (21% O_2_ and 5% CO_2_).

Whole-cell lysates were prepared in ice-cold Igepal lysis containing: 10 mM Tris (pH 7.6), 0.25 M NaCl and 0.5% Igepal/NP-40. Each sample was loaded into an acrylamide gel along with a protein ladder (Thermo Scientific, UK). The same antibodies were used as for the tissue samples.

### Statistical analysis

All data are presented as means ± SEM unless otherwise stated. Differences were tested using an ANOVA test completed with a post hoc Bonferroni test. Statistical analyses were performed using the IBM SPSS Statistics software package (version 22). Statistical significance was considered as *p*-value < 0.05.

## Results

### HIF1A expression in human AT

**Fig. 1:**
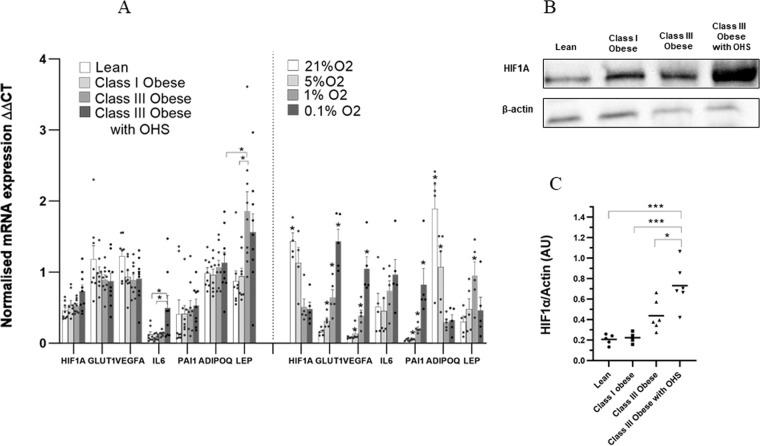
HIF1A expression profiling. **A** mRNA expression of HIF1A, GLUT1, VEGFA, IL6, PAI1, ADIPOQ and LEP in AT from lean (mean BMI = 24.2 kg/m_2_, *n* = 8), class I obese (mean BMI = 32.5 kg/m_2_, *n* = 8), class III obese (mean BMI = 45.3 kg/m_2_, *n* = 9), and class III obese with OHS (mean BMI = 45.3 kg/m_2_, *n* = 9) subjects was determined by real-time qPCR. Data are shown as ΔΔCT values (normalised to PPIA and UBC; mean ± SEM). mRNA expression of HIF1A, GLUT1, VEGFA, IL6, PAI1, ADIPOQ and LEP in mature adipocytes after treatment with different O2 levels was determined by real-time qPCR. Data are shown as ΔΔCT values (normalised to PGK1; *n* = 5, mean ± SEM). **B** Protein expression of HIF1A in the whole AT from lean (*n* = 5), class I obese (*n* = 4), class III obese (*n* = 6), and class III obese with obesity hypoventilation syndrome (*n* = 6) subjects was determined by western blot. **C** Protein densitometry of HIF1A in the four distinct phenotypes. Densitometry data were normalised to β-actin (*<0.05, **<0.001, ***<0.0001).

AT gene expression of *HIF1A* and hypoxia-sensitive genes were assessed in the four distinct groups: lean, class I, class III obese individuals with and without OHS. There were no statistically significant differences in *HIF1A* mRNA levels between lean, class I and class III obese individuals (Fig. 1A). However, *HIF1A* mRNA expression was higher in the class III obese subjects with OHS versus lean controls (*p* = 0.02). AT HIF1A protein content showed a graded response between the groups with the highest abundance in the class III obese subjects with OHS, whereas the class I obese group showed no difference with lean participants (Fig. 1B, C). Class I obese individuals showed significantly higher fasting plasma insulin concentrations compared to lean controls (*p* = 0.03), but without change in fasting glucose levels (Table 1).

### Transcriptomic consequences of hypoxia activation in human AT

There were no significant differences in expression of well-established hypoxia-sensitive genes, including *HIF1A*, *GLUT1*, *VEGFA*, *PAI1* or *ADIPOQ* in AT biopsies between the four groups (Fig. 1A). However, there was a significantly higher expression of *IL6* in the class III obese subjects with OHS versus lean (*p* = 0.04) and class I obese controls (*p* = 0.02), while *LEP* was significantly higher in class III obese subjects compared to lean (*p* = 0.02) and class I obese controls (*p* = 0.04; Fig. 1A).

### Cellular markers of hypoxia in cultured human primary adipocytes

Cellular HIF1A protein expression response to hypoxia (0.1%, 1% or 5% O_2_) seemed graded where all hypoxic levels showed a response. However, again, the mRNA levels were paradoxical for HIF1A, very clear and consistent for a few direct HIF1A targets and mixed for other hypoxia-related targets indicating that the transcriptome is an unreliable read-out for hypoxia.

## Discussion

AT hypoxia has been suggested as a possible cause of AT dysfunction in obesity and type 2 diabetes [10]. There is clear evidence that obesity in rodents results in hypoxic AT, as demonstrated by direct measurements of AT PO_2_ [11, 12]. Low O_2_ availability could potentially shift metabolic patterns towards glycolysis or lactate accumulation, but we did not observe this in a more recent study of whole body adipose tissue in vivo comparing lean and obese subjects [1]. However, studies in humans aiming at directly quantifying hypoxia or tissue PO_2_ resulted in conflicting data suggesting normoxia/hyperoxia [4] or hypoxia [3] in obese individuals. This discrepancy may be explained at least in part by heterogeneity in the study population as well as the different techniques used for the determination of hypoxia. In addition, these studies measured AT hypoxia/PO_2_ in only one place in the tissue. Therefore, the possibility that hypoxic areas may exist in certain parts of the abdominal subcutaneous AT depot in obese subjects cannot be excluded.

The premise of our study is that HIF1A protein abundance in AT is a valid read-out for the presence of hypoxia. The stabilised HIF1A subunit regulates a well-defined network driving an adaptive cellular response to hypoxia [13]. HIF1A is arguably a natural definition of tissue hypoxia with its direct transcriptional regulation of metabolism and vascularisation.

We first measured AT expression of *HIF1A* at the mRNA level in all four groups, observing no significant differences between lean, class I and class III obese subjects. This is consistent with an earlier study comparing lean and obese individuals [14], but in contrast to data from Cancello et al. [15] who showed higher *HIF1A* mRNA expression in morbidly obese subjects versus lean controls. Neither of these studies provide any information on actual AT hypoxia whereas the design of our study was such that we quantified systemic hypoxia in our class III obese subjects. Also, our in vitro studies of hypoxia in human adipocytes showed a paradoxical reduction in the *HIF1A* transcript whereas the downstream targets sometimes showed expected directional effects. This dissociation between transcript and protein is potentially explained by the stabilised HIF1A subunit indirectly promoting *HIF1A* mRNA degradation under sustained hypoxic conditions [16]. Consequently, *HIF1A* mRNA appears to poorly reflect actual hypoxia whereas the presence of HIF1A protein represents a valid marker.

The quantification of AT HIF1A protein abundance comparing lean and class I obese subjects showed no difference, despite class I obese having signs of metabolic dysregulation (significantly higher insulin resistance values and lower HDL). However, there was a clear increase in AT HIF1A protein abundance in class III obesity with actual hypoventilation and an intermediate response in class III obesity without hypoventilation.

In conclusion, these results argue that a genuine hypoxic response in AT is present in severe obesity, in particular if combined with systemic hypoxia (OHS), while it would not support the notion that a hypoxic response drives AT dysfunction at lower levels of obesity.
